# A Para-Ovarian Cyst Infected With Salmonella: A Case Report

**DOI:** 10.7759/cureus.104066

**Published:** 2026-02-22

**Authors:** Vasu Vashishtha, Brij B Agarwal, Chandra Mansukhani, Siddharth Gilda, Ayush Mishra

**Affiliations:** 1 Laparoscopic and General Surgery, Sir Ganga Ram Hospital, New Delhi, IND; 2 Obstetrics and Gynecology, Sir Ganga Ram Hospital, New Delhi, IND; 3 General Surgery, Sir Ganga Ram Hospital, New Delhi, IND; 4 Laparoscopic, Laser and General Surgery, Sir Ganga Ram Hospital, New Delhi, IND

**Keywords:** dermoid cyst, para-ovarian cyst, pyrexia of unknown origin, right iliac fossa mass, salmonella typhi

## Abstract

Extraintestinal Salmonella infections are rare, even in ovarian cysts, and a Salmonella-infected para-ovarian dermoid cyst has not been reported in the literature. A 19-year-old unmarried girl was admitted with a complaint of high-grade continuous fever that was non-amenable to antipyretics. Evaluation revealed leukocytosis with negative blood and stool cultures and positive IgM levels for Salmonella. Imaging showed a large pelvic mass with mixed fat densities, focal calcification, fluid in the pelvis, and patchy fluorodeoxyglucose (FDG) avidity abutting both ovaries and a few pelvic lymph nodes. A lower midline laparotomy was performed, revealing a large pelvic mass separate from the right ovary, which was excised while preserving the ovary. Tufts of hair, focal calcification, and free-flowing pus were seen inside the mass. Examination showed a dermoid with Salmonella infection. We report the first case of Salmonella infection of a para-ovarian dermoid cyst. Extraintestinal manifestations of Salmonella are known, but ovarian involvement is rare, and para-ovarian involvement has not been reported. We highlight the importance of an extensive search for a cause with blood investigations and imaging to reach a conclusive diagnosis. Even in the absence of positive blood and stool cultures, Salmonella can cause infection at unusual sites. Imaging studies may not differentiate between ovarian and para-ovarian cysts, and an infection may mimic a neoplasm on PET scan. Exploration should be performed with excision of the para-ovarian mass, preserving the ovaries when possible.

## Introduction

Para-ovarian cysts arise from mesonephric (Wolffian) duct remnants within the broad ligament and constitute approximately 5%-20% of adnexal masses, often remaining clinically indistinguishable from ovarian lesions [1]. Para-ovarian dermoid cysts are not commonly reported in the literature. Mature cystic teratomas (dermoid cysts) are germ cell tumors composed of well-differentiated ectodermal elements that may act as a nidus for secondary infection. Salmonella is primarily an organism causing enteric infections amenable to antibiotic therapy. Extraintestinal salmonellosis with S. typhi is uncommon, accounting for approximately 5%-10% of all Salmonella infections. The most frequent presentation outside the gut is bacteremia, and when focal organ involvement occurs, the reticuloendothelial system, particularly the liver and spleen, along with bones, especially in individuals with hemoglobinopathies, the breast [2], and very rarely the ovaries, are involved [3,4]. Salmonella infection of a para-ovarian dermoid cyst has not been reported in the literature.

## Case presentation

**Figure 1:**
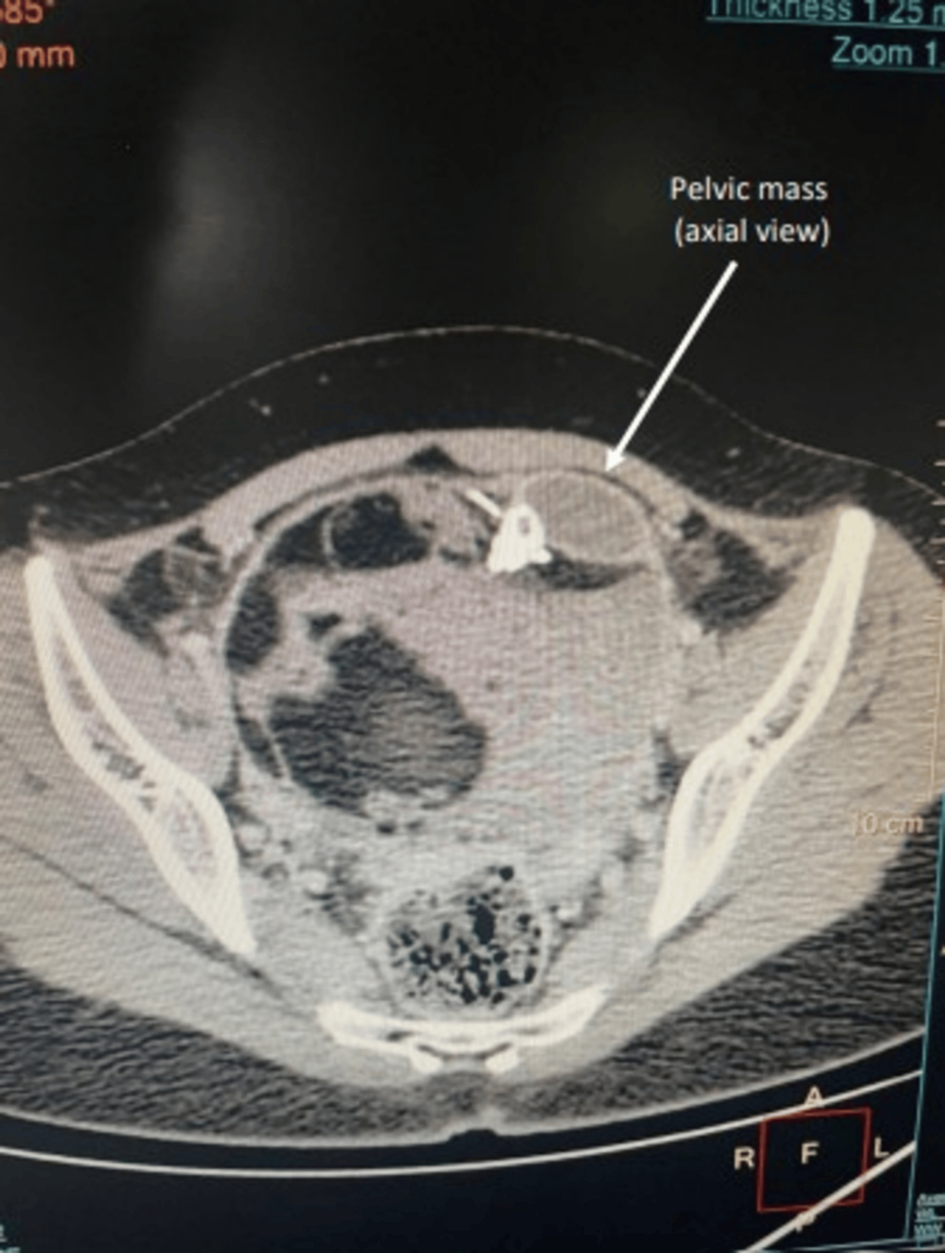
Radiographic contrast-enhanced computed tomography pelvis locating the pelvic mass (axial view) with the 112 x 98 x 118 mm well-defined heterogeneous lesion in focus. White arrow marks the pelvic mass in axial view.

**Figure 2:**
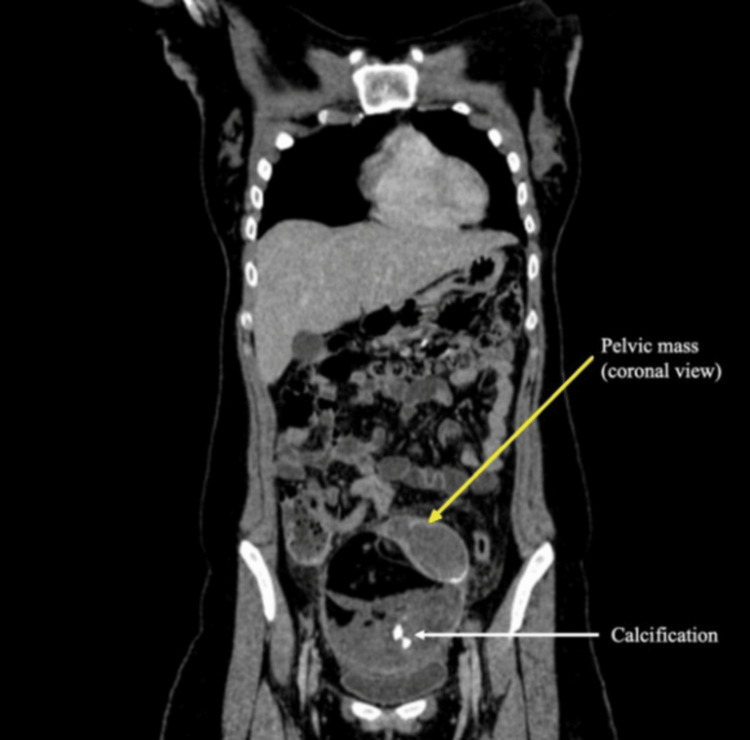
Radiographic contrast-enhanced computed tomography pelvis (coronal section) locating the pelvic mass of size 112 x 98 x 118 mm with area of internal calcification. The yellow arrow indicates the pelvic mass, and the white arrow indicates the calcification.

A 19-year-old female student presented to our department with a history of fever for one week that was non-amenable to antipyretics and not responding to broad-spectrum antibiotics prescribed by a local general physician. The fever was continuous, with rising peaks associated with chills and not returning to normal temperature even with antipyretics. There was no history of abdominal pain, loose stools, or burning micturition. She is an unmarried girl with regular menstrual cycles. She presented to the outpatient department walking, was well hydrated, and was able to communicate about her disease in an articulate manner. She was febrile (38.3°C) at the time of examination, with no tachycardia. She had a red spot rash over the abdomen; otherwise, her general physical examination was normal. On per abdominal examination, a 6 × 6 cm non-tender, smooth-surfaced, globular mass was palpable in the right iliac fossa, with the lower margin not palpable. A working diagnosis of pyrexia of unknown origin (PUO) with a right iliac fossa mass was made, and she was further evaluated. Her laboratory tests revealed leukocytosis with raised absolute monocyte count and absolute neutrophil count, along with raised ESR in the first hour and C-reactive protein assay (CRP). Her liver function, thyroid profile, and renal function tests were normal (Table 1). In view of PUO, extensive investigations were performed to rule out potential causes. Her peripheral smear for malarial parasites, dengue, scrub typhus, brucella, and hydatid serology was negative. Her blood, urine, and stool cultures were negative. Quantiferon TB Gold test was also negative. Inflammatory markers such as ferritin and rheumatoid factor were negative. Tumor markers, including alpha-fetoprotein, lactate dehydrogenase (LDH), cancer antigen 15-3 (CA 15-3), and beta-human chorionic gonadotropin (b-HCG), were all negative. Her IgM for Salmonella typhi was positive, whereas IgG was negative. Multiple imaging modalities were used to identify the cause of fever. Her chest X-ray was normal. Ultrasound of the whole abdomen revealed a lesion in the right adnexa with complex echogenicity, anterosuperior to the urinary bladder, with mild vascularity. Contrast-enhanced CT scan showed a large pelvic mass (112 × 98 × 118 mm) abutting both ovaries, with mixed fat densities, focal calcification, and fluid in the pelvis, along with a few pelvic lymph nodes (Figures 1, 2). 

**Table 1:** Summary of laboratory findings of the patient at admission highlighting inflammatory markers and Salmonella serology.

Parameter	Patient Value	Reference Range
Hemoglobin	11 g/dL	12–15 g/dL
Total leukocyte count	14.15 x 10^3^/µL	4–11 x 10^3^/µL
Absolute neutrophil count	10,330/µL	2000–7000/µL
Absolute monocyte count	1557/µL	200–1000/µL
ESR (1st hour)	105 mm	<20 mm
C-reactive protein	50 mg/L	<5 mg/L
Liver function tests	Normal	Normal
Renal function tests	Normal	Normal
Salmonella typhi IgM	Positive	Negative
Salmonella typhi IgG	Negative	Negative
Blood culture	Negative	Negative
Stool culture	Negative	Negative

In view of the above findings, intravenous ceftriaxone 2 g twice a day was started along with supportive and resuscitative management. Despite 48 hours of intravenous antimicrobial therapy, her fever continued to peak.

**Figure 3:**
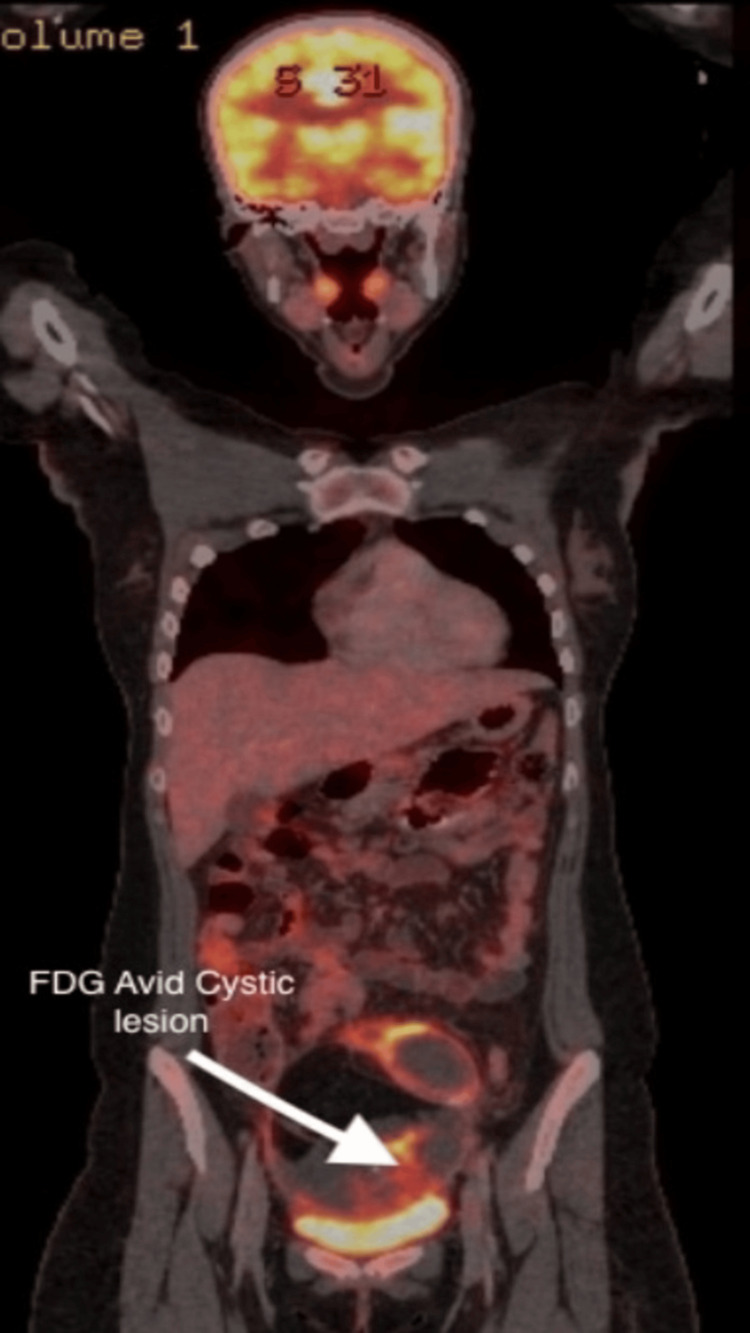
PET-CT scan of the whole body, coronal section, showing an FDG-avid lesion in the pelvis. The white arrow indicates a cystic lesion of the para-ovarian site. PET-CT: positron emission tomography–computed tomography, FDG: fluorodeoxyglucose.

A PET scan was ordered in view of the heterogeneous nature of the tumor and its large size, which showed a large, lobulated, well-defined soft tissue mass lesion in the pelvis with various areas of fluorodeoxyglucose (FDG) avidity, fat necrosis, fat-attenuating lesions, and hyperdense content within multiple enhancing FDG-avid septae. FDG-avid aortocaval lymph nodes were reported (Figure 3).

After a multidisciplinary team discussion, a decision for exploratory laparotomy was made to avoid spillage of the contents of the pelvic mass. A lower midline laparotomy was performed. All quadrants of the abdomen were examined, and no collection or abnormality was detected. A 15 × 9 × 13 cm mass was seen in the right adnexa, separate from the right ovary, within the broad ligament. Preserving the right ovary, the para-ovarian mass was carefully excised using an endo GI stapler gun.

**Figure 4:**
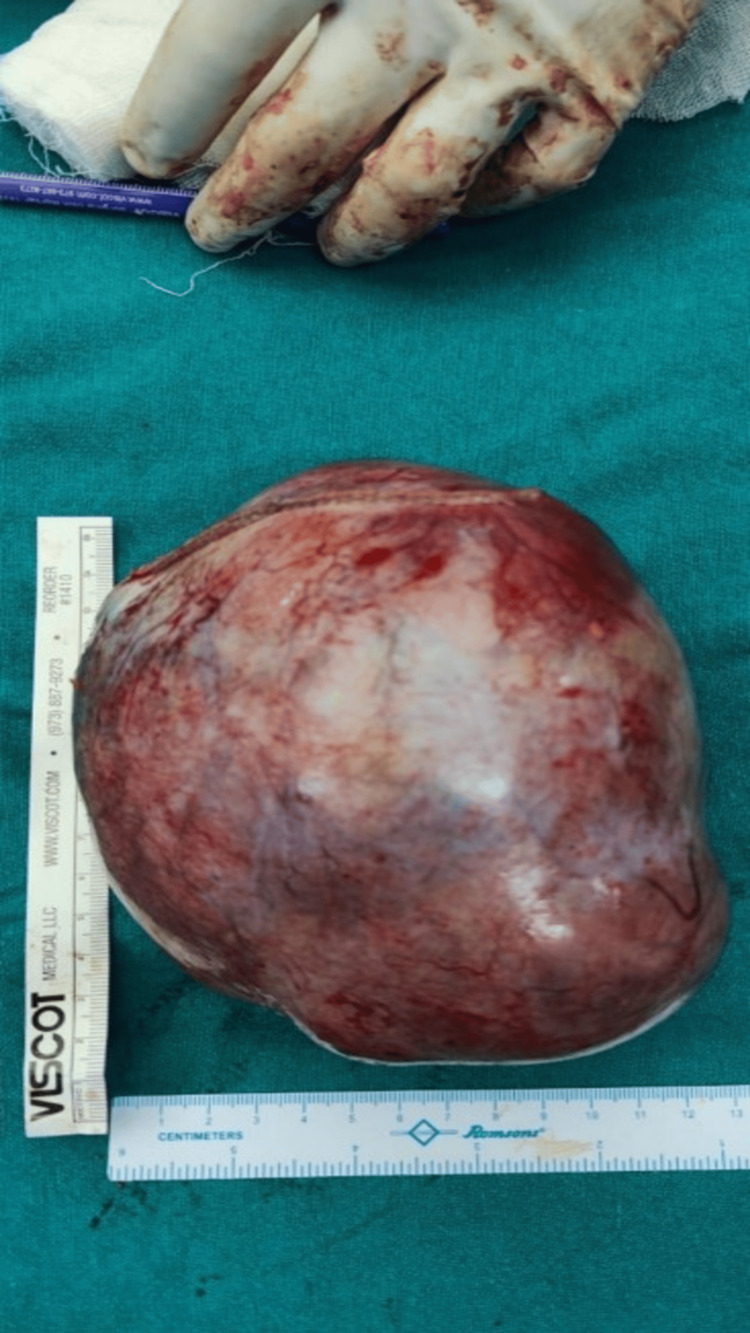
Excised specimen measuring approximately 12 × 15 cm, showing purulent content with hair tufts and calcified elements consistent with a mature cystic teratoma.

The mass was cut open and showed a foul-smelling, purulent collection within it, intermixed with tufts of hair and areas of calcification, along with solid and cystic areas (Figure 4).

The pus was sent for culture and sensitivity, and the rest of the mass was sent for histopathological examination. There was dense growth of Salmonella typhi on pus culture, sensitive to cephalosporins, beta-lactams, azithromycin, and piperacillin. She was started on intravenous Augmentin 1.25 g thrice daily for two days, followed by oral medication in the ward. Postoperatively, she had no fever, started an oral diet on postoperative day one, and began ambulating. She was discharged on postoperative day four on oral medications in a stable condition. The histopathology showed a mature cystic teratoma with inflammation.

## Discussion

Salmonella species can cause both intestinal and extraintestinal infections. The different manifestations depend on virulence and host factors such as weakened immunity and diseased tissue. Extraintestinal infections are far less common than intestinal complications [4]. Salmonella ovarian abscesses occur very rarely and, when they occur, they are usually associated with a preexisting ovarian pathology such as endometrioma, dermoid, or ovarian cyst [5]. Upon extensive review of the literature, 15 cases of superinfection of ovarian cysts with Salmonella have been reported from 1963 to 2019; three of these occurred in pregnant females [6-8]. The preexisting ovarian conditions included endometrioma, dermoid, or simple cyst. Five of these cases had tubal involvement; the rest involved only the ovary [4,9-18]. Salmonella infection in the body can present with fever, abdominal pain, diarrhea, anorexia, or even none of these symptoms. The presence of a Salmonella-infected ovarian cyst is very rarely reported in the literature we reviewed; even rarer is the presence of an infected para-ovarian cyst. There are no reported cases of a Salmonella-infected para-ovarian dermoid cyst based on our review. Para-ovarian cysts constitute only 5%-20% of all adnexal masses, and their complications range from torsion, hemorrhage, rupture, and enlargement to benign tumors [18,19]. Infection of a para-ovarian cyst has not yet been documented. Management of a para-ovarian cyst depends on age, presentation, size, and associated complications. Para-ovarian cysts cannot be clinically distinguished from ovarian cysts, and even on ultrasonographic study, it is difficult to differentiate between the two. There are no specific guidelines on how to manage a para-ovarian cyst; however, when infected, it requires excision, and in a patient with future fertility considerations, an attempt should be made to excise the adnexal mass while preserving the ovaries. In our case, the patient was a teenage, sexually non-active female with no preexisting ovarian pathology who presented with fever not amenable to antibiotics and antipyretics and with no digestive symptoms, along with a palpable adnexal mass. The only clue toward Salmonella infection was positive IgM levels; both stool and blood cultures were negative. This shows that hematogenous spread cannot be ruled out on the basis of blood culture alone, and Salmonella infection cannot be excluded in the absence of digestive symptoms. Imaging studies showed a pelvic mass that could not be differentiated from an ovarian mass, and PET showed FDG avidity. Intraoperative findings made it clear that the mass was well away from the ovaries and fallopian tubes, because of which it was possible to save them, which is imperative in an unmarried female of reproductive age, and it was reported to be a dermoid cyst with culture showing Salmonella infection.

## Conclusions

This is a rare case of a Salmonella para-ovarian abscess and, to our knowledge, the only one reported in the literature. It had unique diagnostic and management challenges. It is important to recognize that Salmonella infections can have extraintestinal manifestations even in the absence of typical symptoms. Imaging or cultures alone could not definitively determine the diagnosis; a comprehensive teamwork approach is essential. Even advanced imaging such as PET scans can overdiagnose, potentially misleading treatment. Early intervention in such cases, with removal of the culprit lesion while preserving essential organs, should be followed. Antibiotics alone may not be sufficient when a confined source of infection is present; hence, pus culture and histopathological diagnosis complete the circle of treatment that started with the search for clues. Preserving fertility remains important by sparing healthy ovaries.
